# Biosynthesis, Metabolism and Function of Auxin, Salicylic Acid and Melatonin in Climacteric and Non-climacteric Fruits

**DOI:** 10.3389/fpls.2019.00136

**Published:** 2019-02-18

**Authors:** Marina Pérez-Llorca, Paula Muñoz, Maren Müller, Sergi Munné-Bosch

**Affiliations:** ^1^Department of Evolutionary Biology, Ecology and Environmental Sciences, University of Barcelona, Barcelona, Spain; ^2^Institute for Research on Nutrition and Food Safety, University of Barcelona, Barcelona, Spain

**Keywords:** auxin, biotrophs, defenses, fruit ripening, maturation, melatonin, post-harvest, salicylates

## Abstract

Climacteric and non-climacteric fruits are differentiated by the ripening process, in particular by the involvement of ethylene, high respiration rates and the nature of the process, being autocatalytic or not, respectively. Here, we focus on the biosynthesis, metabolism and function of three compounds (auxin, salicylic acid and melatonin) sharing not only a common precursor (chorismate), but also regulatory functions in plants, and therefore in fruits. Aside from describing their biosynthesis in plants, with a particular emphasis on common precursors and points of metabolic diversion, we will discuss recent advances on their role in fruit ripening and the regulation of bioactive compounds accumulation, both in climacteric and non-climacteric fruits.

## Introduction

Correct progression of fruit ripening is essential to achieve both optimal fruit quality and long shelf life, important traits that determine price markets and final profits. However, ripening is a complex process where many factors are involved, including hormonal control, which regulates biochemical and physiological changes that give final organoleptic and nutritional fruit properties. Traditionally, fleshy fruits have been classified according to there ripening process into climacteric and non-climacteric fruits. Climacteric fruits such as tomatoes or bananas require an upsurge in respiration rate and ethylene production to unleash the ripening process in an autocatalytic response (Barry and Giovannoni, 2007; Liu et al., 2015). Antagonistically, non-climacteric fruits like citrus fruits or grapes do not exhibit a burst neither in ethylene nor respiration prior to ripening onset, and in these fruits, ripening is mainly controlled by progressive accumulation of the phytohormone abscisic acid (ABA; Rodrigo et al., 2006; Castellarin et al., 2011). Nevertheless, new studies are reinforcing the idea that the ripening process is not only governed by the production of one phytohormone but rather seeming to be the result of a controlled hormonal balance (Symons et al., 2012; Teribia et al., 2016; Li et al., 2019).

Auxins, salicylic acid (SA), and melatonin are phytohormones involved in the signaling and regulation of many crucial processes in plants. Auxins have been widely described as growth and development regulators with multiple functions in plants (see Taylor-Teeples et al., 2016); SA triggers the defense response against biotrophic and hemi-biotrophic pathogens (Loake and Grant, 2007) as well as having an important role under abiotic stress (Dong et al., 2014; Wani et al., 2017), flowering and cell cycle control (Carswell et al., 1989; Eberhard et al., 1989, respectively); and melatonin has not only been found to have auxin-like functions (Chen et al., 2009; Zuo et al., 2014; Wen et al., 2016) but it also has been suggested to act as a potential antioxidant in some plants (Arnao and Hernández-Ruiz, 2015) and a regulator of plant responses to pathogens (Chen et al., 2018). Interestingly, these three hormones share a common precursor – chorismate -, thus a metabolic cross-talk occurs between them, and a number of genes must be finely regulated to divert chorismate metabolism toward these compounds. During the past few years, several reviews have addressed the biosynthesis and role of auxin (Paul et al., 2012), SA (Asghari and Aghdam, 2010) and melatonin (Feng et al., 2014; Arnao and Hernández-Ruiz, 2018) in fruits. However, their biosynthesis and functions have mostly been described separately. Here, we will discuss the common and differential aspects of the biosynthesis, metabolism and function of auxin, SA and melatonin in the growth and ripening of climacteric and non-climacteric fruits. An emphasis will be put on metabolic diversion key points in their biosynthesis from chorismate, the regulatory role during the ripening of climacteric and non-climacteric fruits, and their role in the modulation of the biosynthesis of bioactive compounds, which largely determines fruit quality.

## Chorismate-Derived Phytohormones

**FIGURE 1:**
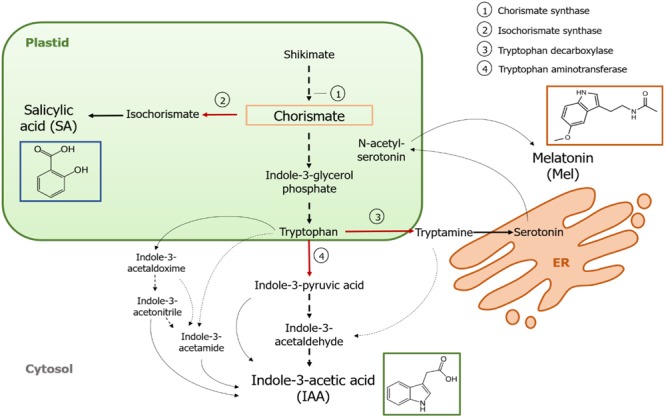
Biosynthesis of indole-3-acetic acid (IAA), salicylic acid (SA), and melatonin (Mel) from chorismate. All three compounds share a common final precursor (chorismate, squared in orange) from the shikimate pathway. Major diversion points are indicated with red arrows and the corresponding enzymes are numbered in a descent divergence order from shikimate. Bold dashed arrows indicate more than one step between compounds. Thin dashed arrows indicate pathways that are still not well defined.

Chorismate is the final product of the shikimate pathway and plays a key role in the biosynthesis of phytohormones (Figure 1). Chorismate gives rise to aromatic amino acids, including tryptophan, and through several reactions, including the conversion of tryptophan to indole-3-pyruvic acid by the tryptophan aminotransferase (TA); it can generate the auxin indole-3-acetic acid (IAA). TA has been proposed as a universal key enzyme to IAA biosynthesis not only in vegetative organs (Enríquez-Valencia et al., 2018) but also in the development of reproductive organs (Reyes-Olalde et al., 2017), including fruit growth and ripening (Estrada-Johnson et al., 2017). By contrast, when isochorismate synthase (ICS) is activated, chorismate can be converted into isochorismate and the latter can be transformed into SA. Despite the fact that this pathway was first identified in bacteria, it is also currently well established in plants (Wildermuth et al., 2001; Uppalapati et al., 2007; Catinot et al., 2008; Garcion et al., 2008; Abreu and Munné-Bosch, 2009). Finally, tryptophan can turn into tryptamine by the enzyme tryptophan decarboxylase (TDC). Although this is another of the multiple routes of IAA biosynthesis (tryptamine turns into indole-3-acetaldehyde and later into IAA), TDC has also been proposed to be the first rate-limiting enzyme in the melatonin pathway (Kang et al., 2007). TDC was first identified in the Apocynaceae family (De Luca et al., 1989) to be later described in a number of plant systems (Byeon et al., 2012, 2014; Zhao et al., 2013; Wei et al., 2018). After decarboxylation, tryptamine is converted into serotonin by the tryptamine-5-hydroxylase, and then serotonin transforms into N-acetyl-serotonin to finally yield melatonin in the cytosol (Figure 1).

Key-diverting enzymes that lead to the synthesis of the chorismate-pathway hormones have been recently identified in fleshy fruits (Figure 1). *ICS*, which expression has been shown to be crucial for biotic stress tolerance (Garcion et al., 2008; Zeng and He, 2010), has been identified in tomatoes and apples. Zhu et al. (2016) reported the overexpression of *ICS1* (*isochorismate synthase 1*) under cold storage conditions in tomato, while Zhang et al. (2017) found activation of a transcription factor involved in the pathogen-related SA signaling pathway inducing *ICS* expression in apples. Enhanced TDC activity has been reported to occur in unripe pepper fruit upon infection by pathogens – through increased *TDC1* and *TDC2* expression (Park et al., 2013) – and in the growth stage in mulberry fruit (Wang et al., 2016). Finally, TA has been identified in grapevine, both at *pre-* and *véraison* (Böttcher et al., 2013; Gouthu and Deluc, 2015, respectively) as well as during strawberry ripening (Estrada-Johnson et al., 2017). It is noteworthy, however, that the genes and enzymes described in climacteric fruits (i.e., ICS) have not been identified in non-climacteric fruits (i.e., TDC and TA) and vice versa; hence, further studies are imperative to fill these knowledge gaps and better understand how these diversion points are jointly regulated during fruit ripening.

## Role of Chorismate-Derived Phytohormones in Climacteric and Non-Climacteric Fruits

Unraveling the mechanisms of fruit development has been one of the major challenges in recent agronomy research for its economic implications. In this context, phytohormones have been pointed out as accountable drivers of fruit ripening, specially ethylene and ABA in climacteric and non-climacteric fruits, respectively. However, that these phytohormones could regulate fruit development alone was soon proven to be far too simple. After extensive research and with the improvement in analytical chemistry and molecular techniques, several other hormones have been confirmed as potential regulators of fruit development and ripening, including chorismate-derived phytohormones.

### Auxins Cross-Talk With Other Hormones During Fruit Set, Growth and Ripening

**FIGURE 2:**
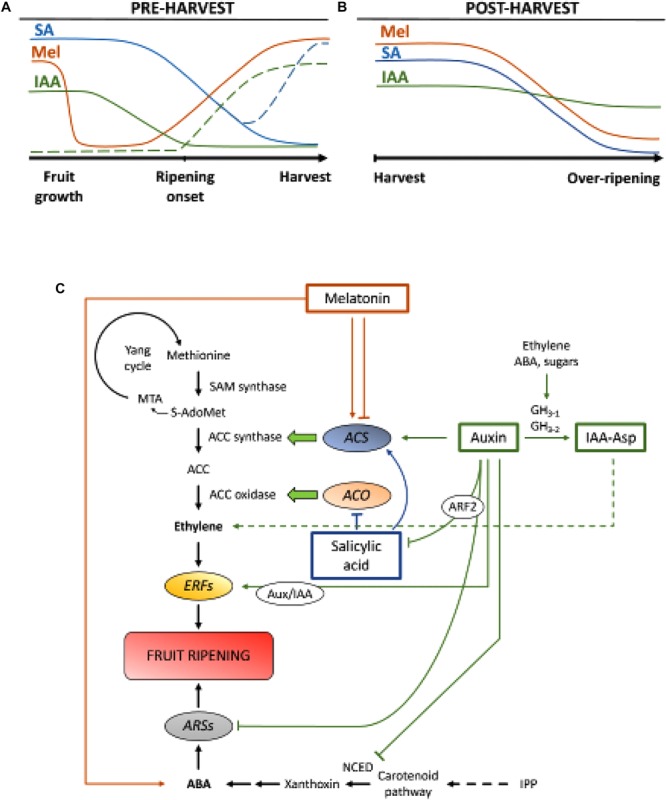
Role of IAA, SA, and Mel during the development of climacteric and non-climacteric fruits. Model summarizing the interactions of IAA, SA, and Mel during the ripening of climacteric and non-climacteric fruits during **(A)** pre- and **(B)** post-harvest. Dashed lines indicate alternative dynamics of phytohormone contents in some fruits (see text for discussion). **(C)** Overview of the interaction of IAA, SA, and Mel with ethylene and abscisic acid (ABA) biosynthesis in climacteric and non-climacteric fruits. Auxin is a positive regulator of ethylene biosynthesis by the activation of ACC synthase genes (*ACS*) and inducing the expression of Ethylene Responsive Factors (ERFs). Auxin also represses ABA production by *9-cis-epoxycarotenoid dioxygenase* (*NCED*) repression and inhibiting ABA-responsive stress genes (*ARS*) by the ARF2 protein. Endogenous IAA contents can be reduced by GH_3_ proteins, the synthesis of which is promoted by ethylene, ABA and sugars. Discontinuous line indicates the possible role of IAA by IAA-Asp as a ripening factor enhancing ethylene in some fruits. Melatonin enhances ABA and ethylene production, although ACS can be inhibited depending on melatonin contents. SA acts as an inhibitor of ethylene repressing ACC oxidase (*ACO*) genes, although it can promote the expression of *ACS*. *MTA, 50-methylthioadenosine; S-AdoMet, S-adenosyl-methionine; SAM synthase, S-adenosyl-methionine synthase; ACC, 1-aminocyclopropane-1-carboxylic acid; IPP, isopentenyl diphosphate*.

Auxins are a group of plant hormones that play an essential role in fruit development, both exerting their own influence and modulating expression of other phytohormones. Endogenous contents of IAA are particularly high at fruit set and during initial growth developmental stages, after which IAA amounts tend to decline before ripening onset, both in climacteric (Zaharah et al., 2012) and non-climacteric fruits (Symons et al., 2012; Teribia et al., 2016), with apparently some exceptions, like peaches (Tatsuki et al., 2013) and some plum varieties (El-Sharkawy et al., 2014; Figure 2A). It has been demonstrated that IAA is involved in fruit set initiation in combination with gibberellins (Mezzetti et al., 2004; Serrani et al., 2010; Bermejo et al., 2018; Hu et al., 2018). Impairment of IAA biosynthesis or signaling generally leads to fruit parthenocarpy, although it may also result in abnormal ripening in some fruits (Wang et al., 2005; Liu J. et al., 2018; Reig et al., 2018). High contents of IAA at initial stages of fruit development promote fruit growth due to auxin implication in cell division in combination with cytokinins and in the control of cell expansion in combination with gibberellins (Liao et al., 2018). During this period, hormonal crosstalk between auxins and gibberellins additionally allows normal fruit shaping in a fine-tuned regulation mediated by Auxin Response Factors (ARFs; Liao et al., 2018; Liu S. et al., 2018).

Reductions of endogenous IAA contents have been reported to occur before the onset of ripening in several fruits. These reductions have been related to IAA conjugation with aspartic acid (IAA-Asp) by IAA-amido synthetases, GH_3-1_ and GH_3-2_ (Figure 2C). Indeed, *GH_3-1_ and GH_3-2_* showed higher expression during early fruit development and most particularly during ripening initiation both in climacteric fruits, such as tomatoes (Sravankumar et al., 2018) and apples (Onik et al., 2018), as well as in non-climacteric fruits, like grape berries (Böttcher et al., 2010, 2011) and raspberries (Bernales et al., 2019). Interestingly, grape berries showed enhanced *GH_3-1_* expression after ABA and ethephon application, which could explain the involvement of ethylene in the control of IAA contents after the onset of ripening, even in non-climacteric fruits (Böttcher et al., 2010). In fact, several studies highlight the tight interaction between auxins and ethylene in fruit ripening, with a reciprocal influence between them (Tadiello et al., 2016a; Busatto et al., 2017). For climacteric fruits, increased contents of IAA are necessary to activate expression of ACC synthase genes (*ACS*), encoding ACC synthase, which, in turn, will lead to ethylene production triggering the ripening process (Figure 2C; Tatsuki et al., 2013). Fruits like tomatoes (Li et al., 2016) or bananas (Choudhury et al., 2008), which usually experience reduced IAA contents prior to the onset of ripening, show a ripening delay when IAA or analogs are exogenously applied, while fruits like peaches, whose IAA contents increase progressively until fully ripen, show accelerated ripening when auxins are applied (Tadiello et al., 2016b). Therefore, a tight, complex and differential regulation of auxin-ethylene interactions must exist in various fruits during preharvest ripening. Furthermore, IAA contents decrease before ripening onset in some non-climacteric fruits to de-repress *9-cis-epoxycarotenoid dioxygenase* (*NCED*) and start ABA synthesis (Figure 2C; Jia et al., 2016). Interestingly, this process is also mediated by IAA conjugation through enhanced GH_3-1_ activity (Böttcher et al., 2010). Nevertheless, Estrada-Johnson et al. (2017) showed that, although IAA contents decrease during strawberry ripening, expression of *FaAux/IAA* and *FaARF* gene families are induced in red receptacles, suggesting the involvement of auxin signaling in fully ripen fruits.

During post-harvest, auxin contents usually remain invariant or tend to decrease due to oxidative processes that may give rise to small, but progressive reductions in the endogenous contents of IAA (Figure 2B). Auxin treatments after harvest delay over-ripening in some fruits (Chen et al., 2016; Moro et al., 2017) and increase the contents of some organic acids, maintaining fruit acidity (Li et al., 2017), thus suggesting auxin also plays a significant role in the control of fruit ripening during post-harvest.

### Multiple Roles of Salicylic Acid During Fruit Development and Ripening

Salicylic acid is another chorismate-derived phytohormone that has been mostly related to its protective effect under biotic stress to control preharvest and post-harvest losses derived from pathogen fruit infection (Babalar et al., 2007; Cao et al., 2013). In general, endogenous contents of free SA are higher at the beginning of fruit development and then decrease progressively (Oikawa et al., 2015). Lu et al. (2015) reported a secondary increase in SA during the second growth phase of peach fruits. Exogenous application of SA in bananas delayed the ripening process reducing the respiratory burst and the pulp to peel ratio, as well as decreasing activity of enzymes related to cell wall degradation and antioxidant system (Srivastava and Dwivedi, 2000). Moreover, treatment of sweet cherry trees with salicylic and acetylsalicylic acid (ASA) enhanced weight, firmness and color of cherries at commercial stage (Giménez et al., 2014). Exogenous application of SA or metyl salicylate (MeSA) during post-harvest, delayed over-ripening in fruits like kiwis (Zhang et al., 2003), and sweet cherries (Valero et al., 2011). Furthermore, treatments with salicylic or ASA alleviated chilling injury of pomegranates (Sayyari et al., 2011), tomatoes (Aghdam et al., 2014), and avocados (Glowacz et al., 2017). SA also interacts with ethylene through inhibition of ACC oxidase (*ACO*) expression (Shi and Zhang, 2012). Surprisingly, increased contents of *ACS* expression in pears are also found after SA application in a dose-regulated manner (Shi et al., 2013; Shi and Zhang, 2014). Therefore, regulation of ethylene production dependent on SA is finely regulated (Figure 2C).

### Melatonin: An Emerging Regulator for the Control of Fruit Development

Melatonin is also a plant hormone from the chorismate-derived pathway that has gained much attention in the recent years (Hernández-Ruiz et al., 2005; Arnao and Hernández-Ruiz, 2018; Sharif et al., 2018). Recently, melatonin has been pointed out as an important factor for fruit set, in a similar way to IAA effects (Figure 2A; Liu S. et al., 2018). Besides, circadian production of this phytohormone has been observed (Kolář et al., 1997). During on-tree ripening, grapes treated with melatonin showed higher synchronicity and increased weight when fully ripens (Meng et al., 2015). Moreover, another recent study by Xu et al. (2018) described that melatonin could promote grape berry ripening through its interaction with ABA and ethylene, as well as hydrogen peroxide. It has been demonstrated that melatonin confers chilling tolerance during fruit cold storage, which appears to be mostly related to its antioxidant activity (Cao et al., 2018; Aghdam et al., 2019). During post-harvest, tomatoes showed increased ethylene emission after application of melatonin at 50 μM (Sun et al., 2015), contrary to pears, where exogenous application of melatonin at 100 μM inhibited ethylene production (Zhai et al., 2018), or banana fruits, where melatonin at 50–200 μM inhibited the expression of *ACS* and *ACO* (Figure 2C; Hu et al., 2017). Although different effects of melatonin application may be related to a dosage effect, further research is needed to better understand the role of melatonin in the control of fruit development.

### Cross-Talk of Chorismate-Derived Phytohormones in the Control of Fruit Development

Hormonal crosstalk between chorismate-derived phytohormones has been shown to occur during fruit development and ripening. Breitel et al. (2016) found that auxins can interact with SA through ARF2 (Figure 2C) and that overexpression of *ARF2* in tomato resulted in lower contents of SA and a significant ripening delay. This indicates that auxins might be limiting SA production during fruit development, which might explain a trade-off between fruit growth (mediated by auxin) and activation of biotic defenses (mediated by SA). Moreover, exogenous application of SA on papaya fruits resulted in an altered expression of several *IAA* genes, some of them being down-regulated while others up-regulated (Liu et al., 2017). In this case, results were inconclusive of what really occurs endogenously in the putative cross-talk between auxin and SA during fruit ripening and further research is required in this and other climacteric fruits. In any case, it is clear that IAA and SA are closely related, not only metabolically but also functionally. Finally, several studies have reported the influence of melatonin on IAA and SA biosynthesis and/or signaling (reviewed in Arnao and Hernández-Ruiz, 2018), although to our knowledge, none of these studies was performed neither in climacteric nor in non-climacteric fruits.

## Modulation of Bioactive Compounds Biosynthesis by Chorismate-Derived Phytohormones

Bioactive compounds (including phenolic compounds, isoprenoids, and antioxidant vitamins) have not only been widely investigated as responsible for specific organoleptic properties of foods, but also for their protective effects in human cells against oxidative processes in the development of neurodegenerative and cardiovascular diseases and certain type of cancer (Liu et al., 1999; Mueller et al., 2010; Sturgeon and Ronnenberg, 2010; Björkman et al., 2011). Phenolic compounds contribute significantly to imparting specific flavors, such as tannins – responsible for the bitterness or astringency taste of certain fruits, and colors, such as anthocyanin pigments – responsible for red, blue and purple fruit colors (Croteau et al., 2000). The relevance of phenolic compounds for human consumption has been associated with a protective effect against oxidative processes in relation to cardiovascular and central nervous system health as well as a reduced risk for cancers of the gastrointestinal tract (Björkman et al., 2011). Carotenoids, tetraterpenes belonging to isoprenoids, play a role in the protection against photo-oxidative processes and as organic pigments; they are responsible for the orange-yellow color of fruits (Tapiero et al., 2004). Dietary carotenoids are thought to provide health benefits in decreasing the risk of eye disease and certain types of cancers due to their role as antioxidants (Johnson, 2002). Vitamins C and E, including ascorbic acid and tocopherols, respectively, are essential nutritional quality factors in fruits with many biological activities in humans (Miret and Müller, 2017). Considering the relevant role of all these bioactive compounds as health promoting compounds in fruits, the modulation of their accumulation is of paramount importance in both climacteric and non-climacteric fruits (Vasconsuelo and Boland, 2007). Most of these bioactive compounds are accumulated at high levels during fruit ripening, when less palatable green fruits converse into a nutritionally rich, colored, and tasty fruit (Daood et al., 1996; Ranalli et al., 1998; Dumas et al., 2003; Singh et al., 2011). However, once commenced, ripening cannot be stalled and generally leads to over-ripening that negatively affects the fruit quality (Kumar et al., 2014). Therefore, minimizing post-harvest fruit spoilage while obtaining high contents of bioactive compounds remains one of the biggest challenges to resolve.

Auxin has been identified as an important regulator of carotenoid biosynthesis during ripening of climacteric tomato fruits. Ripening in tomato is associated with the degradation of chlorophyll and the shift of xanthophylls to carotenes (β-carotene and lycopene) (Fraser et al., 1994). IAA appears to delay tomato ripening by repressing ethylene and several upstream carotenoid transcripts including *Psy, Ziso, Pds, Critiso* as well as *Chlases 1–3* and, on the other hand, promoting the accumulation of β*-Lyc1* and *Crtr-*β*1* transcripts, resulting in higher contents of xanthophylls and chlorophyll a (Su et al., 2015). In addition, in some non-climacteric fruits such as cherry and grape berries auxin also seems to modulate anthocyanin biosynthesis and thereby control fruit ripening processes. Teribia et al. (2016) reported a negative correlation between IAA and anthocyanin contents indicating that anthocyanin accumulation starts when IAA contents decrease. Moreover, the application of the synthetic auxin-like compound benzothiazole-2-oxyacetic acid (BTOA) delayed the up-regulation of genes that promote enzymes of the anthocyanin biosynthesis such as chalcone synthase and UDP-glucose:flavonoid 3-*O*-glucosyltransferase in grape berries (Davies et al., 1997).

Pre- and post-harvest treatments with salicylates, including SA and its derivatives ASA and MeSA, have been reported to regulate bioactive compounds accumulation leading to improved antioxidant activity in both climacteric and non-climacteric fruits. Several fruits such as plum, cherry and apricots are known to have a short life with a rapid deterioration in quality after harvest. Therefore, there is a constant search of treatments that improve and maintain fruit quality, and especially the content of bioactive compounds with beneficial health effects. Treatments of plums with SA, ASA, and MeSA resulted in significant higher contents of ascorbic acid, anthocyanins and phenolic compounds both at harvest and after prolonged cold storage (40 days at 4°C; Martínez-Esplá et al., 2017). Similar results could be observed for preharvest treatments with SA, ASA, and MeSA in sweet cherries (Giménez et al., 2014, 2015, 2017). Furthermore, post-harvest treatment with SA, ASA, or MeSA maintained total phenolic contents as well as anthocyanin contents during cold storage in pomegranate (Sayyari et al., 2011), sweet cherry (Valero et al., 2011), cornelian cherry (Dokhanieh et al., 2013), and apricot (Wang et al., 2015). These results suggest that salicylates may be involved in the activation of phenylalanine ammonia lyase, which is the main enzyme involved in the biosynthesic pathway of phenolic compounds (Martínez-Esplá et al., 2017).

Recently, melatonin has been shown to regulate fruit ripening and modulate bioactive compounds. Post-harvest treatments with melatonin in peach fruits increased chilling tolerance by activating antioxidant systems. Chilling injury occurs in peach fruits during low temperature storage (between 2 and 7.6°C) characterized by flesh browning, abnormal ripening and higher sensitivity to decay (Lurie and Crisosto, 2005). Cao et al. (2018) reported that peaches treated with melatonin showed higher transcript abundance of ascorbic acid biosynthesis genes *PpGME, PpGPP*, and *PpGLDH* at seventh and *PpGMPH* at the 21st and 28th day of storage compared to control resulting in increased ascorbic acid contents. Moreover, melatonin induced increases in activities of G6PDH, SKDH and PAL, which are the essential enzymes for phenolic compounds biosynthesis. The authors suggest that melatonin treatment protects peach fruit to a certain degree from chilling injury by specifically activating the biosynthesis of phenolic compounds (Gao et al., 2018). Additionally, a label-free differential proteomics analysis revealed the effect of melatonin on promoting fruit ripening and anthocyanin accumulation upon post-harvest in tomato fruits (Sun et al., 2016). The authors reported that exogenous melatonin increased eight enzymes related to the anthocyanin pathway including inter alia flavonol-3-hydroxylase, flavanone 3 beta-hydroxylase, anthocyanidin synthase/leucoanthocyanidin dioxygenase, and anthocyanidin 3-*O*-glucosyltransferase. Moreover, post-harvest treatment with melatonin increased total phenols and anthocyanins in strawberry fruits (Aghdam and Fard, 2016), and delayed loss of total phenols, flavonoids and anthocyanins in litchi fruits (Zhang et al., 2018). Treatments with melatonin increased the contents of phenols, anthocyanins and flavonoids in grape berries (Xu et al., 2017). Additionally, melatonin has been reported to enhance lycopene accumulation and ethylene production in tomatoes, suggesting that melatonin may increase the content of lycopene by impacting ethylene biosynthesis and signaling (Sun et al., 2015). The participation of ethylene in the melatonin-induced regulation of bioactive compounds has also been observed in grape berries, where double block treatment of ethylene showed reduced effects of melatonin on polyphenol contents (Xu et al., 2017).

## Conclusion

It is concluded that chorismate-derived phytohormones, including auxin, SA and melatonin, not only share a common precursor but play essential roles in the regulation of fruit growth and ripening. A metabolic and functional cross-talk between them and with other phytohormones occurs in a spatiotemporal manner to finely regulate the development of climacteric and non-climacteric fruits. The differences in the dynamics within climacteric and non-climacteric fruits evidence that the response of chorismate-derived hormones is not universal but rather strongly species-specific. Furthermore, chorismate-derived phytohormones also modulate the accumulation of bioactive compounds, thus influencing fruit quality. Further research is, however, needed to better understand how (i) other hormones, such as ethylene and ABA, modulate their biosynthesis studying key metabolic diversion points, (ii) they interact at the functional and molecular levels, and (iii) they jointly modulate bioactive compounds biosynthesis and consequently influence not only fruit growth and ripening, but also the quality of climacteric and non-climacteric fruits.

## Author Contributions

SM-B conceived and designed the review with the help of MP-L. MP-L, PM, MM, and SM-B wrote the manuscript. MP-L prepared figures with the help of PM. All authors contributed to the discussion of ideas, revised and approved the final manuscript.

## Conflict of Interest Statement

The authors declare that the research was conducted in the absence of any commercial or financial relationships that could be construed as a potential conflict of interest.

## References

[B1] Abreu M. E. Munné-Bosch S. (2009). Salicylic acid deficiency in NahG transgenic lines and sid2 mutants increases seed yield in the annual plant *Arabidopsis thaliana*. *J. Exp. Bot.* 60 1261–1271. 10.1093/jxb/ern363 19188277 PMC2657544

[B2] Aghdam M. S. Asghari M. Khorsandi O. Mohayeji M. (2014). Alleviation of postharvest chilling injury of tomato fruit by salicylic acid treatment. *J. Food Sci. Technol.* 51 2815–2820. 10.1007/s13197-012-0757-1 25328231 PMC4190258

[B3] Aghdam M. S. Fard J. R. (2016). Melatonin treatment attenuates postharvest decay and maintains nutritional quality of strawberry fruits (*Fragaria* x *anannasa* cv. Selva) by enhancing GABA shunt activity. *Food Chem.* 221 1650–1657. 10.1016/j.foodchem.2016.10.123 27979142

[B4] Aghdam M. S. Luo Z. Jannatizadeh A. Sheikh-Assadi M. Sharafi Y. Farmani B. (2019). Employing exogenous melatonin applying confers chilling tolerance in tomato fruits by upregulating ZAT2/6/122/6/12 giving rise to promoting endogenous polyamines, proline, and nitric oxide accumulation by triggering arginine pathway activity. *Food Chem.* 275 549–556. 10.1016/j.foodchem.2018.09.157 30724232

[B5] Arnao M. B. Hernández-Ruiz J. (2015). “Melatonin: synthesis from tryptophan and its role in higher plants,” in *Amino Acids in Higher Plants* ed. D’ Mello J. P. F. (Boston, CA: CAB International) 390–435. 10.1079/9781780642635.0390

[B6] Arnao M. B. Hernández-Ruiz J. (2018). Melatonin and its relationship to plant hormones. *Ann. Bot.* 121 195–207. 10.1093/aob/mcx114 29069281 PMC5808790

[B7] Asghari M. Aghdam M. S. (2010). Impact of salicylic acid on postharvest physiology of horticultural crops. *Trends Food Sci. Technol.* 21 502–509. 10.1016/j.tifs.2010.07.009

[B8] Babalar M. Asghari M. Talaei A. Khosroshahi A. (2007). Effect of pre- and postharvest salicylic acid treatment on ethylene production, fungal decay and overall quality of Selva strawberry fruit. *Food Chem.* 105 449–453. 10.1016/j.foodchem.2007.03.021

[B9] Barry C. S. Giovannoni J. J. (2007). Ethylene and fruit ripening. *J. Plant Growth Regul.* 26 143–159. 10.1007/s00344-007-9002-y

[B10] Bermejo A. Granero B. Mesejo C. Reig C. Tejedo V. Agustí M. (2018). Auxin and gibberellin interact in citrus fruit set. *J. Plant Growth Regul.* 37 491–501. 10.1007/s00344-017-9748-9

[B11] Bernales M. Monsalve L. Ayala-Raso A. Valdenegro M. Martínez J. P. Travisany D. (2019). Expression of two indole-3-acetic acid (IAA)-amido synthetase (GH3) genes during fruit development of raspberry (*Rubus idaeus* Heritage). *Sci. Hortic.* 246 168–175. 10.1016/j.scienta.2018.09.077 30480063

[B12] Björkman M. Klingen I. Birch A. N. Bones A. M. Bruce T. J. Johansen T. J. (2011). Phytochemicals of Brassicaceae in plant protection and human health — Influences of climate, environment and agronomic practice. *Phytochemistry* 72 538–556. 10.1016/j.phytochem.2011.01.014 21315385

[B13] Böttcher C. Boss P. K. Davies C. (2011). Acyl substrate preferences of an IAA-amido synthetase account for variations in grape (*Vitis vinifera* L.) berry ripening caused by different auxinic compounds indicating the importance of auxin conjugation in plant development. *J. Exp. Bot.* 62 4267–4280. 10.1093/jxb/err134 21543520 PMC3153680

[B14] Böttcher C. Burbidge C. A. Boss P. K. Davies C. (2013). Interactions between ethylene and auxin are crucial to the control of grape (*Vitis vinifera* L.) berry ripening. *BMC Plant Biol.* 13:222. 10.1186/1471-2229-13-222 24364881 PMC3878033

[B15] Böttcher C. Keyzers R. A. Boss P. K. Davies C. (2010). Sequestration of auxin by the indole-3-acetic acid-amido synthetase GH3-1 in grape berry (*Vitis vinifera* L.) and the proposed role of auxin conjugation during ripening. *J. Exp. Bot.* 61 3615–3625. 10.1093/jxb/erq174 20581124

[B16] Breitel D. A. Chappell-Maor L. Meir S. Panizel I. Puig C. P. Hao Y. (2016). AUXIN RESPONSE FACTOR 2 intersects hormonal signals in the regulation of tomato fruit ripening. *PLoS Genet.* 12:e1005903. 10.1371/journal.pgen.1005903 26959229 PMC4784954

[B17] Busatto N. Salvagnin U. Resentini F. Quaresimin S. Navazio L. Marin O. (2017). The peach RGF/GLV signaling peptide pCTG134 is involved in a regulatory circuit that sustains auxin and ethylene actions. *Front. Plant Sci.* 11:1711. 10.3389/fpls.2017.01711 29075273 PMC5641559

[B18] Byeon Y. Park S. Kim Y.-S. Park D.-H. Lee S. Back K. (2012). Light-regulated melatonin biosynthesis in rice during the senescence process in detached leaves. *J. Pineal Res.* 5 107–111. 10.1111/j.1600-079X.2012.00976.x 22289080

[B19] Byeon Y. Park S. Lee H. Y. Kim Y. S. Back K. (2014). Elevated production of melatonin in transgenic rice seeds expressing rice tryptophan decarboxylase. *J. Pineal Res.* 56 275–282. 10.1111/jpi.12120 24433490

[B20] Cao J. K. Yan J. Q. Zhao Y. M. Jiang W. B. (2013). Effects of four preharvest foliar sprays with *β*-aminobutyric acid or salicylic acid on the incidence of post-harvest disease and induced defence responses in jujube (*Zizyphus jujuba* Mill.) fruit after storage. *J. Hortic. Sci. Biotechnol.* 88 338–344. 10.1080/14620316.2013.11512974

[B21] Cao S. Shao J. Shi L. Xu L. Shen Z. Chen W. (2018). Melatonin increases chilling tolerance in postharvest peach fruit by alleviating oxidative damage. *Sci. Rep.* 8:806. 10.1038/s41598-018-19363-5 29339757 PMC5770464

[B22] Carswell G. K. Johnson C. M. Shillito R. D. Harms C. T. (1989). O-acetyl-salicylic acid promotes colony formation from protoplasts of an elite maize inbred. *Plant Cell Rep.* 8 282–284. 10.1007/BF00274130 24233226

[B23] Castellarin S. D. Gambetta G. A. Wada H. Shackel K. A. Matthews M. A. (2011). Fruit ripening in *Vitis vinifera*: spatiotemporal relationships among turgor, sugar accumulation, and anthocyanin biosynthesis. *J. Exp. Bot.* 62 4345–4354. 10.1093/jxb/err150 21586429 PMC3153685

[B24] Catinot J. Buchala A. Abou-Mansour E. Métraux J.-P. (2008). Salicylic acid production in response to biotic and abiotic stress depends on isochorismate in *Nicotiana benthamiana*. *FEBS Lett.* 582 473–478. 10.1016/j.febslet.2007.12.039 18201575

[B25] Chen J. Mao L. Lu W. Ying T. Luo Z. (2016). Transcriptome profiling of postharvest strawberry fruit in response to exogenous auxin and abscisic acid. *Planta* 243 183–197. 10.1007/s00425-015-2402-5 26373937

[B26] Chen Q. Qi W. B. Reiter R. J. Wei W. Wang B. M. (2009). Exogenously applied melatonin stimulates root growth and raises endogenous indoleacetic acid in roots of etiolated seedlings of *Brassica juncea*. *J. Plant Physiol.* 166 324–328. 10.1016/j.jplph.2008.06.002 18706737

[B27] Chen X. Sun C. Laborda P. Zhao Y. Palmer I. Fu Z. Q. (2018). Melatonin treatment inhibits the growth of *Xanthomonas oryzae* pv. *oryzaezae*. *Front. Microbiol.* 9:2280. 10.3389/fmicb.2018.02280 30337911 PMC6180160

[B28] Choudhury S. R. Roy S. Sengupta D. N. (2008). Characterization of transcriptional profiles of MA-ACS1 and MA-ACO1 genes in response to ethylene, auxin, wounding, cold and different photoperiods during ripening in banana fruit. *J. Plant Physiol.* 165 1865–1878. 10.1016/j.jplph.2008.04.012 18554749

[B29] Croteau R. Kutchan T. M. Lewis N. G. (2000). “Natural products (secondary metabolites),” in *Biochemistry & Molecular Biology of Plants: Chemistry & Molecular Biology of Plants* eds Buchanan B. Gruissem W. Jones R. (Rockville, MD: ASPP) 1250–1318.

[B30] Daood H. G. Vinkler M. Márkus F. Hebshi A. Biacs P. A. (1996). Antioxidant vitamin content of spice red pepper (paprika) as affected by technological and varietal factors. *Food Chem.* 55 365–372. 10.1016/0308-8146(95)00136-0

[B31] Davies C. Boss P. K. Robinson S. P. (1997). Treatment of grape berries, a nonclimacteric fruit with a synthetic auxin, retards ripening and alters the expression of developmentally regulated genes. *Plant Physiol.* 115 1155–1161. 10.1104/pp.115.3.1155 12223864 PMC158580

[B32] De Luca V. Marineau C. Brisson N. (1989). Molecular cloning and analysis of cDNA encoding a plant tryptophan decarboxylase: comparison with animal dopa decarboxylases. *Proc. Natl. Acad. Sci.U.S.A.* 86 2582–2586. 10.1073/pnas.86.8.2582 2704736 PMC286961

[B33] Dokhanieh A. Y. Aghdam M. S. Fard J. R. Hassanpour H. (2013). Postharvest salicylic acid treatment enhances antioxidant potential of cornelian cherry fruit. *Sci. Hortic.* 154 31–36. 10.1016/j.scienta.2013.01.025

[B34] Dong C. J. Li L. Shang Q. M. Liu X. Y. Zhang Z. G. (2014). Endogenous salicylic acid accumulation is required for chilling tolerance in cucumber (*Cucumis sativus* L.) seedlings. *Planta* 240 687–700. 10.1007/s00425-014-2115-1 25034826

[B35] Dumas Y. Dadomo M. Di Lucca G. Grolier P. (2003). Effects of environmental factors and agricultural techniques on antioxidant content of tomatoes. *J. Sci. Food Agric.* 83 369–382. 10.1002/jsfa.1370

[B36] Eberhard S. Doubrava N. Marfa V. Mohnen D. Southwick A. Darvill A. (1989). Pectic cell wall fragments regulate tobacco thin-cell-layer explant morphogenesis. *Plant Cell* 1 747–755. 10.1105/tpc.1.8.747 12359909 PMC159812

[B37] El-Sharkawy I. Sherif S. M. Jones B. Mila I. Kumar P. P. Bouzayen M. (2014). TIR1-like auxin-receptors are involved in the regulation of plum fruit development. *J. Exp. Bot.* 65 5205–5215. 10.1093/jxb/eru279 24996652 PMC4157706

[B38] Enríquez-Valencia A. J. ázquez-Flota F. A. Ku-Cauich J. R. Escobedo-GarciaMedrano R. M. (2018). Differentially expressed genes during the transition from early to late development phases in somatic embryo of banana (*Musa* spp. AAB group, Silk subgroup) cv. Manzano. *Plant Cell Tissue Organ Cult.* 136 289–302. 10.1007/s11240-018-1514-6

[B39] Estrada-Johnson E. Csukasi F. Pizarro C. M. Vallarino J. G. Kiryakova Y. Vioque A. (2017). Transcriptomic analysis in strawberry fruits reveals active auxin biosynthesis and signaling in the ripe receptacle. *Front. Plant Sci.* 8:889. 10.3389/fpls.2017.00889 28611805 PMC5447041

[B40] Feng X. Wang M. Zhao Y. Han P. Dai Y. (2014). Melatonin from different sources, functional roles, and analytical methods. *Trends Food Sci. Technol.* 37 21–31. 10.1016/j.tifs.2014.02.001

[B41] Fraser P. D. Truesdale M. R. Bird C. R. Schuch W. Bramley P. M. (1994). Carotenoid biosynthesis during tomato fruit development evidence for tissue-specific gene expression. *Plant Physiol.* 105 405–413. 10.1104/pp.105.1.405 12232210 PMC159369

[B42] Gao H. Lu Z. Yang Y. Wang S. Yang T. Cao M. (2018). Melatonin treatment reduces chilling injury in peach fruit through its regulation of membrane fatty acid contents and phenolic metabolism. *Food Chem.* 245 659–666. 10.1016/j.foodchem.2017.10.008 29287423

[B43] Garcion C. Lohmann A. Lamodière E. Catinot J. Buchala A. Doermann P. (2008). Characterization and biological function of the *ISOCHORISMATE SYNTHASE2* gene of Arabidopsis. *Plant Physiol.* 147 1279–1287. 10.1104/pp.108.119420 18451262 PMC2442540

[B44] Giménez M. J. Serrano M. Valverde J. M. Martínez-Romero D. Castillo S. Valero D. (2017). Preharvest salicylic acid and acetylsalicylic acid treatments preserve quality and enhance antioxidant systems during postharvest storage of sweet cherry cultivars. *J. Sci. Food Agric.* 97 1220–1228. 10.1002/jsfa.7853 27312073

[B45] Giménez M. J. Valverde J. M. Valero D. Díaz-Mula H. M. Zapata P. J. Serrano M. (2015). Methyl salicylate treatments of sweet cherry trees improve fruit quality at harvest and during storage. *Sci. Hortic.* 197 665–673. 10.1016/j.scienta.2015.10.033

[B46] Giménez M. J. Valverde J. M. Valero D. Guillén F. Martínez-Romero D. Serrano M. (2014). Quality and antioxidant properties on sweet cherries as affected by preharvest salicylic and acetylsalicylic acids treatments. *Food Chem.* 160 226–232. 10.1016/j.foodchem.2014.03.107 24799232

[B47] Glowacz M. Bill M. Tinyane P. P. Sivakumar D. (2017). Maintaining postharvest quality of cold stored ‘Hass’ avocados by altering the fatty acids content and composition with the use of natural volatile compounds – methyl jasmonate and methyl salicylate. *J. Sci. Food. Agric.* 97 5186–5193. 10.1002/jsfa.8400 28447342

[B48] Gouthu S. Deluc L. G. (2015). Timing of ripening initiation in grape berries and its relationship to seed content and pericarp auxin levels. *BMC Plant Biol.* 15:46. 10.1186/s12870-015-0440-6 25848949 PMC4340107

[B49] Hernández-Ruiz J. Cano A. Arnao M. B. (2005). Melatonin acts as a growth-stimulating compound in some monocot species. *J. Pineal Res.* 39 137–142. 10.1111/j.1600-079X.2005.00226.x 16098090

[B50] Hu J. Israeli A. Ori N. Suna T. P. (2018). The Interaction between DELLA and ARF/IAA mediates crosstalk between gibberellin and auxin signaling to control fruit initiation in tomato. *Plant Cell* 30 1710–1728. 10.1105/tpc.18.00363 30008445 PMC6139683

[B51] Hu W. Yang H. Tie W. Yan Y. Ding Z. Liu Y. (2017). Natural variation in banana varieties highlights the role of melatonin in postharvest ripening and quality. *J. Agric. Food Chem.* 65 9987–9994. 10.1021/acs.jafc.7b03354 29077394

[B52] Jia H. Jiu S. Zhang C. Wang C. Tariq P. Liu Z. (2016). Abscisic acid and sucrose regulate tomato and strawberry fruit ripening through the abscisic acid-stress-ripening transcription factor. *Plant Biotechnol. J.* 14 2045–2065. 10.1111/pbi.12563 27005823 PMC5043491

[B53] Johnson E. J. (2002). The role of carotenoids in human health. *Nutr. Clin. Carer* 5 56–65. 10.1046/j.1523-5408.2002.00004.x 12134711

[B54] Kang S. Kang K. Lee K. Back K. (2007). Characterization of rice tryptophan decarboxylases and their direct involvement in serotonin biosynthesis in transgenic rice. *Planta* 227 263–272. 10.1007/s00425-007-0614-z 17763868

[B55] Kolář J. Macháčková I. Eder J. Prinsen E. van Dongen W. van Onckelen H. (1997). Melatonin: occurrence and daily rhythm in *Chenopodium rubrum*. *Phytochemistry* 44 1407–1413. 10.1016/S0031-9422(96)00568-7

[B56] Kumar R. Khurana A. Sharma A. K. (2014). Role of plant hormones and their interplay in development and ripening of fleshy fruits. *J. Exp. Bot.* 65 4561–4575. 10.1093/jxb/eru277 25028558

[B57] Li J. Khan Z. U. Taoa X. Maoa L. Luoa Z. Yinga T. (2017). Effects of exogenous auxin on pigments and primary metabolite profile of postharvest tomato fruit during ripening. *Sci. Hortic.* 219 90–97. 10.1016/j.scienta.2017.03.011

[B58] Li J. Tao X. Li L. Mao L. Luo Z. Khan Z. U. (2016). Comprehensive RNA-Seq analysis on the regulation of tomato ripening by exogenous auxin. *PLoS One* 11:e0156453. 10.1371/journal.pone.0156453 27228127 PMC4881990

[B59] Li Y. Lua Y. Lia L. Chu Z. Zhang H. Li H. (2019). Impairment of hormone pathways results in a general disturbance of fruit primary metabolism in tomato. *Food Chem.* 274 170–179. 10.1016/j.foodchem.2018.08.026 30372923

[B60] Liao X. Li M. Liu B. Yan M. Yu X. Zi H. (2018). Interlinked regulatory loops of ABA catabolism and biosynthesis coordinate fruit growth and ripening in woodland strawberry. *Proc. Natl. Acad. Sci. U.S.A.* 115 E11542–E11550. 10.1073/pnas.1812575115 30455308 PMC6298082

[B61] Liu J. Zhai R. Liu F. Zhao Y. Wang H. Liu L. (2018). Melatonin induces parthenocarpy by regulating genes in gibberellin pathways of ‘Starkrimson’ Pear (*Pyrus communis* L.). *Front. Plant Sci.* 9:946. 10.3389/fpls.2018.00946 30022992 PMC6040045

[B62] Liu S. Zhang Y. Feng Q. Qin L. Pan C. Lamin-Samu A. T. (2018). Tomato AUXIN RESPONSE FACTOR 5 regulates fruit set and development via the mediation of auxin and gibberellin signaling. *Sci. Rep.* 8:2971. 10.1038/s41598-018-21315-y 29445121 PMC5813154

[B63] Liu K. Yuan C. Feng S. Zhong S. Li H. Zhong J. (2017). Genome-wide analysis and characterization of *Aux/IAA* family genes related to fruit ripening in papaya (*Carica papaya* L.). *BMC Genomics* 18:351. 10.1186/s12864-017-3722-6 28476147 PMC5420106

[B64] Liu M. Pirrello J. Chervin C. Roustan J.-P. Bouzayen M. (2015). Ethylene control of fruit ripening: revisiting the complex network of transcriptional regulation. *Plant Physiol.* 169 2380–2390. 10.1104/pp.15.01361 26511917 PMC4677914

[B65] Liu S. Stampfer M. J. Hu F. B. Giovannucci E. Rimm E. Manson J. E. (1999). Whole-grain consumption and risk of coronary heart disease: results from the nurses’ health study. *Am. J. Clin. Nutr.* 70 412–419. 10.1093/ajcn/70.3.412 10479204

[B66] Loake G. Grant M. (2007). Salicylic acid in plant defence—the players and protagonists. *Curr. Opin. Plant Biol.* 10 466–472. 10.1016/j.pbi.2007.08.008 17904410

[B67] Lu Q. Zhang W. Gao J. Lu M. Zhang L. Li J. (2015). Simultaneous determination of plant hormones in peach based on dispersive liquid-liquid microextraction coupled with liquid chromatography-ion trap mass spectrometry. *J. Chromatogr. B Analyt. Technol. Biomed. Life Sci.* 992 8–13. 10.1016/j.jchromb.2015.04.014 25939092

[B68] Lurie S. Crisosto C. H. (2005). Chilling injury in peach and nectarine. *Postharvest Biol. Technol.* 37 195–208. 10.1016/j.postharvbio.2005.04.012

[B69] Martínez-Esplá A. Zapata P. J. Valero D. Martínez-Romero D. Díaz-Mula H. M. Serrano M. (2017). Preharvest treatments with salicylates enhance nutrient and antioxidant compounds in plum at harvest and after storage. *J. Sci. Food Agric.* 98 2742–2750. 10.1002/jsfa.8770 29105771

[B70] Meng J. F. Xu T. F. Song C. Z. Yu Y. Hu F. Zhang L. (2015). Melatonin treatment of pre-veraison grape berries to increase size and synchronicity of berries and modify wine aroma components. *Food Chem.* 185 127–134. 10.1016/j.foodchem.2015.03.140 25952850

[B71] Mezzetti B. Landi L. Pandolfini T. Spena A. (2004). The defH9-iaaM auxin-synthesizing gene increases plant fecundity and fruit production in strawberry and raspberry. *BMC Biotechnol.* 4:4. 10.1186/1472-6750-4-4 15113427 PMC394336

[B72] Miret J. A. Müller M. (2017). “AsA/DHA redox pair influencing plant growth and stress tolerance,” in *Ascorbic Acid in Plant Growth, Development and Stress Tolerance Ascorbic Acid in Plant Growth, Development and Stress Tolerance* eds Hossain M. Munné-Bosch S. Burritt D. Diaz-Vivancos P. Fujita M. Lorence A. (Cham: Springer) 297–319.

[B73] Moro L. Aymoto N. M. Purgatto E. (2017). Postharvest auxin and methyl jasmonate effect on anthocyanin biosynthesis in red raspberry (*Rubus idaeus* L.). *J. Plant Growth. Regul.* 36 773–782. 10.1007/s00344-017-9682-x

[B74] Mueller M. Hobiger S. Jungbauer A. (2010). Anti-inflammatory activity of extracts from fruits, herbs and spices. *Food Chem.* 122 987–996. 10.1016/j.foodchem.2010.03.041

[B75] Oikawa A. Otsuka T. Nakabayashi R. Jikumaru Y. Isuzugawa K. Murayama H. (2015). Metabolic profiling of developing pear fruits reveals dynamic variation in primary and secondary metabolites, including plant hormones. *PLoS One* 10:e0131408. 10.1371/journal.pone.0131408 26168247 PMC4500446

[B76] Onik J. C. Hu X. Lin Q. Wang Z. (2018). Comparative transcriptomic profiling to understand pre- and post-ripening hormonal regulations and anthocyanin biosynthesis in early ripening apple fruit. *Molecules* 31:E1908. 10.3390/molecules23081908 30065188 PMC6222687

[B77] Park S. Le T.-N. N. Byeon Y. Kim Y. S. Back K. (2013). Transient induction of melatonin biosynthesis in rice (*Oryza sativa* L.) during reproductive stage. *J. Pineal Res.* 55 40–45. 10.1111/jpi.12021 23110463

[B78] Paul V. Pandey R. Srivastava G. C. (2012). The fading distinctions between classical patterns of ripening in climacteric and non-climacteric fruit and the ubiquity of ethylene—An overview. *J. Food Sci. Technol.* 49 1–21. 10.1007/s13197-011-0293-4 23572821 PMC3550874

[B79] Ranalli A. Tombesi A. Ferrante M. L. De Mattia G. (1998). Respiratory rate of olive drupes during their ripening cycle and quality of oil extracted. *J. Sci. Food Agric.* 77 359–367. 10.1002/(SICI)1097-0010(199807)77:3<359::AID-JSFA43>3.0.CO;2-R

[B80] Reig C. Martínez-Fuentes A. Mesejo C. Agustí M. (2018). Hormonal control of parthenocarpic fruit set in ‘Rojo Brillante’ persimmon (*Diospyros kaki* Thunb.). *J. Plant Physiol.* 238 96–104. 10.1016/j.jplph.2018.09.004 30248556

[B81] Reyes-Olalde J. I. Zúñiga-Mayo V. M. Serwatowska J. Chavez Montes R. A. Lozano-Sotomayor P. Herrera-Ubaldo H. (2017). The bHLH transcription factor SPATULA enables cytokinin signaling, and both activate auxin biosynthesis and transport genes at the medial domain of the gynoecium. *PLoS Genet.* 13:e1006726. 10.1371/journal.pgen.1006726 28388635 PMC5400277

[B82] Rodrigo M. J. Alquezar B. Zacarías L. (2006). Cloning and characterization of two 9-*cis*-epoxycarotenoid dioxygenase genes, differentially regulated during fruit maturation and under stress conditions, from orange (*Citrus sinensis* L. Osbeck). *J. Exp. Bot.* 57 633–643. 10.1093/jxb/erj048 16396998

[B83] Sayyari M. Castillo S. Valero D. Díaz-Mula H. M. Serrano M. (2011). Acetylsalicylic acid alleviates chilling injury and maintains nutritive and bioactive compounds and antioxidant activity during postharvest storage of pomegranates. *Postharvest Biol. Technol.* 60 136–142. 10.1016/j.postharvbio.2010.12.012

[B84] Serrani J. C. Carrera E. Ruiz-Rivero O. Gallego-Giraldo L. Pereira L. E. García-Martínez J. L. (2010). Inhibition of auxin transport from the ovary or from the apical shoot induces parthenocarpic fruit-set in tomato mediated by gibberellins. *Plant Physiol.* 153 851–862. 10.1104/pp.110.155424 20388661 PMC2879769

[B85] Sharif R. Xie C. Zhang H. Arnao M. B. Ali M. Ali Q. (2018). Melatonin and its effects on plant systems. *Molecules* 23:E2352. 10.3390/molecules23092352 30223442 PMC6225270

[B86] Shi H. Wang Y. Qui A. Zhang Y. Xu J. Wang A. (2013). PpACS1b, a pear gene encoding ACC synthase, is regulated during fruit late development and involved in response to salicylic acid. *Sci. Hortic.* 164 602–609. 10.1016/j.scienta.2013.09.055

[B87] Shi H. Y. Zhang Y. X. (2012). Pear *ACO* genes encoding putative 1-aminocyclopropane-1-carboxylate oxidase homologs are functionally expressed during fruit ripening and involved in response to salicylic acid. *Mol. Biol. Rep.* 39 9509–9519. 10.1007/s11033-012-1815-5 22711312

[B88] Shi H. Y. Zhang Y. X. (2014). Expression and regulation of pear 1-aminocyclopropane-1-carboxylic acid synthase gene (PpACS1a) during fruit ripening, under salicylic acid and indole-3-acetic acid treatment, and in diseased fruit. *Mol. Biol. Rep.* 41 4147–4154. 10.1007/s11033-014-3286-3 24562629

[B89] Singh R. K. Ali S. A. Nath P. Sane V. A. (2011). Activation of ethylene-responsive hydroxyphenylpyruvate dioxygenase leads to increased tocopherol levels during ripening in mango. *J. Exp. Bot.* 62 3375–3385. 10.1093/jxb/err006 21430290 PMC3130165

[B90] Sravankumar T. Akash Naik N. Kumar R. (2018). A ripening-induced SlGH3-2 gene regulates fruit ripening via adjusting auxin-ethylene levels in tomato (*Solanum lycopersicum* L.). *Plant Mol. Biol.* 98 455–469. 10.1007/s11103-018-0790-1 30367324

[B91] Srivastava K. M. Dwivedi U. N. (2000). Delayed ripening of banana fruit by salicylic acid. *Plant Sci.* 158 87–96. 10.1016/S0168-9452(00)00304-6 10996248

[B92] Sturgeon S. R. Ronnenberg A. G. (2010). Pomegranate and breast cancer: possible mechanisms of prevention. *Nutr. Rev.* 68 122–128. 10.1111/j.1753-4887.2009.00268.x 20137057

[B93] Su L. Diretto G. Purgatto E. Danoun S. Zouine M. Li Z. (2015). Carotenoid accumulation during tomato fruit ripening is modulated by the auxin-ethylene balance. *BMC Plant Biol.* 15:114. 10.1186/s12870-015-0495-4 25953041 PMC4424491

[B94] Sun Q. Zhang N. Wang J. Cao Y. Li X. Zhang H. (2016). A label-free differential proteomics analysis reveals the effect of melatonin on promoting fruit ripening and anthocyanin accumulation upon postharvest in tomato. *J Pineal Res.* 61 138–153. 10.1111/jpi.12315 26820691

[B95] Sun Q. Zhang N. Wang J. Zhang H. Li D. Shi J. (2015). Melatonin promotes ripening and improves quality of tomato fruit during postharvest life. *J. Exp. Bot.* 66 657–668. 10.1093/jxb/eru332 25147270 PMC4321535

[B96] Symons G. M. Chua Y. J. Ross J. J. Quittenden L. J. Davies N. W. Reid J. B. (2012). Hormonal changes during non-climacteric ripening in strawberry. *J. Exp. Bot.* 63 695–709. 10.1093/jxb/ers147 22791823 PMC3428006

[B97] Tadiello A. Longhi S. Moretto M. Ferrarini A. Tononi P. Farneti B. (2016a). Interference with ethylene perception at receptor level sheds auxin light on fruit transcriptional circuits associated with the climacteric ripening of apple (*Malus x domestica* Borkh.). *Plant J.* 88 963–975. 10.1111/tpj.13306 27531564

[B98] Tadiello A. Ziosi V. Negri A. S. Noferini M. Fiori G. Busatto N. (2016b). On the role of ethylene, auxin and a GOLVEN-like peptide hormone in the regulation of peach ripening. *BMC Plant Biol.* 16:44. 10.1186/s12870-016-0730-7 26863869 PMC4750175

[B99] Tapiero H. Townsend D. M. Tew K. D. (2004). The role of carotenoids in the prevention of human pathologies. *Biomed. Pharmacother.* 58 100–110. 10.1016/j.biopha.2003.12.006 14992791 PMC6361147

[B100] Tatsuki M. Nakajima N. Fujii H. Shimada T. Nakano M. Hayashi K. (2013). Increased levels of IAA are required for system 2 ethylene synthesis causing fruit softening in peach (*Prunus persica* L. Batsch). *J. Exp. Bot.* 64 1049–1059. 10.1093/jxb/ers381 23364941 PMC3580816

[B101] Taylor-Teeples M. Lanctot A. Nemhauser J. L. (2016). As above, so below: auxin’s role in lateral organ development. *Dev. Biol.* 419 156–164. 10.1016/j.ydbio.2016.03.020 26994944 PMC5026869

[B102] Teribia N. Tijero V. Munné-Bosch S. (2016). Linking hormonal profiles with variations in sugar and anthocyanin contents during the natural development and ripening of sweet cherries. *N. Biotechnol.* 33 824–833. 10.1016/j.nbt.2016.07.015 27475901

[B103] Uppalapati S. R. Ishiga Y. Wangdi T. Kunkel B. N. Anand A. Mysore K. S. (2007). The phytotoxin coronatine contributes to pathogen fitness and is required for suppression of salicylic acid accumulation in tomato inoculated with *Pseudomonas syringae* pv. tomato DC3000. *Mol. Plant Microbe Interact.* 20 955–965. 10.1094/MPMI-20-8-0955 17722699

[B104] Valero D. Díaz-Mula H. M. Zapata P. J. Castillo S. Guillén F. Martínez-Romero D. (2011). Postharvest treatments with salicylic acid, acetylsalicylic acid or oxalic acid delayed ripening and enhanced bioactive compounds and antioxidant capacity in sweet cherry. *J. Agric. Food Chem.* 59 5483–5489. 10.1021/jf200873j 21506518

[B105] Vasconsuelo A. Boland R. (2007). Molecular aspects of the early stages of elicitation of secondary metabolites in plants. *Plant Sci.* 172 861–875. 10.1016/j.plantsci.2007.01.006

[B106] Wang C. Yin L.-Y. Shi X.-Y. Xiao H. Kang K. Liu X.-Y. (2016). Effect of cultivar, temperature, and environmental conditions on the dynamic change of melatonin in Mulberry fruit development and wine fermentation. *J. Food Sci.* 81 M958–M967. 10.1111/1750-3841.13263 26953927

[B107] Wang H. Jones B. Li Z. Frasse P. Delalande C. Regad F. (2005). The tomato Aux/IAA transcription factor IAA9 is involved in fruit development and leaf morphogenesis. *Plant Cell* 17 2676–2692. 10.1105/tpc.105.033415 16126837 PMC1242265

[B108] Wang Z. Ma L. Zhang X. Xu L. Cao J. Jiang W. (2015). The effect of exogenous salicylic acid on antioxidant activity, bioactive compounds and antioxidant system in apricot fruit. *Sci. Hortic.* 181 113–120. 10.1016/j.scienta.2014.10.055

[B109] Wani A. B. Chadar H. Wani A. H. Singh S. Upadhyay N. (2017). Salicylic acid to decrease plant stress. *Enviorn. Chem. Lett.* 15 101–123. 10.1007/s10311-016-0584-0

[B110] Wei Y. Liu G. Chang T. Lin D. Reiter R. J. He C. (2018). Melatonin biosynthesis enzymes recruit WRKY transcription factors to regulate melatonin accumulation and transcriptional activity on W-box in cassava. *J. Pineal Res.* 65:e12487. 10.1111/jpi.12487 29528508

[B111] Wen D. Gong B. Sun S. Liu S. Wang X. Wei M. (2016). Promoting roles of melatonin in adventitious root development of *Solanum lycopersicum* L. by regulating auxin and nitric oxide signaling. *Front. Plant Sci.* 7:718. 10.3389/fpls.2016.00718 27252731 PMC4879336

[B112] Wildermuth M. C. Dewdney J. Wu G. Ausubel F. M. (2001). Isochorismate synthase is required to synthesize salicylic acid for plant defence. *Nature* 414 562–565. 10.1038/35107108 11734859

[B113] Xu L. Yue Q. Bian F. Sun H. Zhai H. Yao Y. (2017). Melatonin enhances phenolics accumulation partially via ethylene signaling and resulted in high antioxidant capacity in grape berries. *Front. Plant Sci.* 8:1426. 10.3389/fpls.2017.01426 28868058 PMC5563355

[B114] Xu L. Yue Q. Xiang G. Bian F. Yao Y. (2018). Melatonin promotes ripening of grape berry via increasing the levels of ABA, H2O2, and particularly ethylene. *Hortic. Res.* 5:41. 10.1038/s41438-018-0045-y 30083356 PMC6068098

[B115] Zaharah S. S. Singh Z. Symons G. M. Reid J. B. (2012). Role of brassinosteroids, ethylene, abscisic acid, and indole-3- acetic acid in mango fruit ripening. *J. Plant Growth Regul.* 31 363–372. 10.1007/s00344-011-9245-5

[B116] Zeng W. He S. Y. (2010). A prominent role of the flagellin receptor FLAGELLIN-SENSING2 in mediating stomatal response to *Pseudomonas syringae* pv tomato DC3000 in Arabidopsis. *Plant Physiol.* 153 1188–1198. 10.1104/pp.110.157016 20457804 PMC2899927

[B117] Zhai R. Liu J. Liu F. Zhao Y. Liu L. Fang C. (2018). Melatonin limited ethylene production, softening and reduced physiology disorder in pear (*Pyrus communis* L.) fruit during senescence. *Postharvest Biol. Technol.* 139 38–46. 10.1016/j.postharvbio.2018.01.017

[B118] Zhang H. Chen L. Zhao L. Zheng X. Yang Q. Zhang X. (2017). Investigating proteome and transcriptome defence response of apples induced by *Yarrowia lipolytica*. *Mol. Plant Microbe Interact.* 30 301–311. 10.1094/MPMI-09-16-0189-R 28398122

[B119] Zhang Y. Chen K. Zhang S. Ferguson I. (2003). The role of salicylic acid in postharvest ripening of kiwifruit. *Postharvest Biol. Technol.* 28 67–74. 10.1016/S0925-5214(02)00172-2

[B120] Zhang Y. Huber D. J. Hu M. Jiang G. Gao Z. Xu X. (2018). Delay of postharvest browning in litchi fruit by melatonin via the enhancing of antioxidative processes and oxidation repair. *J. Agric. Food Chem.* 66 7475–7484. 10.1021/acs.jafc.8b0192 29953220

[B121] Zhao Y. Tan D. X. Lei Q. Chen H. Wang L. Li Q. (2013). Melatonin and its potential biological functions in the fruits of sweet cherry. *J. Pineal Res.* 55 79–88. 10.1111/jpi.12044 23480341

[B122] Zhu Z. Ding Y. Zhao J. Nie Y. Zhang Y. Sheng J. (2016). Effects of postharvest gibberellic acid treatment on chilling tolerance in cold-stored tomato (*Solanum lycopersicum* L.) fruit. *Food Bioproc. Tech.* 9 1202–1209. 10.1007/s11947-016-1712-3

[B123] Zuo B. Zheng X. He P. Wang L. Lei Q. Feng C. (2014). Overexpression of MzASMT improves melatonin production and enhances drought tolerance in transgenic *Arabidopsis thaliana* plants. *J. Pineal Res.* 57 408–417. 10.1111/jpi.1218 25250844

